# Characterization and assessment of lung and bone marrow derived endothelial cells and their bone regenerative potential

**DOI:** 10.3389/fendo.2022.935391

**Published:** 2022-08-31

**Authors:** Mariana Moraes de Lima Perini, Conner R. Valuch, Ushashi C. Dadwal, Olatundun D. Awosanya, Sarah L. Mostardo, Rachel J. Blosser, Adam M. Knox, Anthony C. McGuire, Hanisha L. Battina, Murad Nazzal, Melissa A. Kacena, Jiliang Li

**Affiliations:** ^1^ Department of Biology, Indiana University Purdue University Indianapolis, Indianapolis, IN, United States; ^2^ Department of Orthopaedic Surgery, Indiana University School of Medicine, Indianapolis, IN, United States; ^3^ Richard L. Roudebush Veterans Affairs (VA) Medical Center, Indianapolis, IN, United States

**Keywords:** aging, fracture healing, endothelial progenitor cells, angiogenesis, mouse

## Abstract

Angiogenesis is important for successful fracture repair. Aging negatively affects the number and activity of endothelial cells (ECs) and subsequently leads to impaired bone healing. We previously showed that implantation of lung-derived endothelial cells (LECs) improved fracture healing in rats. In this study, we characterized and compared neonatal lung and bone marrow-derived endothelial cells (neonatal LECs and neonatal BMECs) and further asses3sed if implantation of neonatal BMECs could enhance bone healing in both young and aged mice. We assessed neonatal EC tube formation, proliferation, and wound migration ability *in vitro* in ECs isolated from the bone marrow and lungs of neonatal mice. The *in vitro* studies demonstrated that both neonatal LECs and neonatal BMECs exhibited EC traits. To test the function of neonatal ECs *in vivo*, we created a femoral fracture in young and aged mice and implanted a collagen sponge to deliver neonatal BMECs at the fracture site. In the mouse fracture model, endochondral ossification was delayed in aged control mice compared to young controls. Neonatal BMECs significantly improved endochondral bone formation only in aged mice. These data suggest BMECs have potential to enhance aged bone healing. Compared to LECs, BMECs are more feasible for translational cell therapy and clinical applications in bone repair. Future studies are needed to examine the fate and function of BMECs implanted into the fracture sites.

## Introduction

Aging and diseases such as osteoporosis and diabetes are linked to increased bone fragility, resulting in the bone becoming more prone to complications such as fractures (1–4). Most fractures heal naturally, but despite improved surgical techniques, approximately 10% of fractures result in delayed healing or non-unions (5–9). Adequate blood supply appears to be one of the most important factors for proper fracture healing (7, 10). For this reason, increasing blood supply through angiogenesis enhancement is often used in fracture treatment (11). Enhancing angiogenesis improves the transportation of oxygen, cells, and key nutrients to the fracture site (4).

Blood vessels are composed of lining cells known as ECs (12). Circulating endothelial progenitor cells (EPCs) are increased during fracture repair (4, 13). EPCs can promote angiogenesis and improve osteogenesis and bone reconstruction (14). Some of the major origin sites for EPCs are peripheral blood, umbilical cord blood, bone marrow, and tissue-resident cells in vasculature including lungs (11, 15–17). This study will consider cells from two different subtypes, lung derived endothelial cells (LECs) and bone marrow endothelial cells (BMECs).

EPCs are prime candidates for tissue repair, as they can form tube-like networks *in vitro*. They can be directly incorporated into damaged vasculature or initiate vasculogenesis (11, 18–21). In a previous study, LECs were added to induced compound fractures in rats resulting in fractures that displayed better healing when compared to the controls, which exhibited incomplete callus bridging (11). BMECs help give rise to new blood vessels by inducing vasculogenesis as well as osteogenesis and have been shown to aid ischemic tissue repair, including fracture defects (14, 22). They are thought to be circulating angiogenic cells, moving freely through the bone marrow and are recruited when needed (23). The angiogenic potential and the bone repair abilities of EPCs have been studied extensively in healthy animals, especially in rats (11, 24–27). However, it is not clear if EPCs isolated from mice have similar osteogenic potential in bone fracture settings in young and aged mice. From the perspective of future clinical applications, study of BMECs as opposed to LECs has more translational potential.

Improving blood flow to the fracture site may accelerate the rate of healing. Impaired fracture healing in elderly can be attributed to diminished osteogenesis and angiogenesis (28). With aging, EPCs become less active and more prone to undergoing apoptosis (29). Thus, the *in vivo* ability of neonatal BMECs to affect bone repair in a murine femur fracture model was investigated in this study. The endothelial traits of neonatal BMECs and LECs *in vitro* were also compared. Compared to LECs, BMECs are more feasible for translational cell therapy and clinical applications in bone repair. Here, we investigated the potential of neonatal BMECs to aid in fracture repair of aged mice with a femur fracture.

## Methods and materials

### Animal models

All animal procedures were reviewed and approved by the Indiana University School of Medicine Institutional Animal Care and Use Committee (IACUC). Young (3-4 months old) and old (24-26 months old) male C57BL/6J mice were provided by the National Institute on Aging.

In preparation for surgery, mice were anesthetized with isoflurane (Patterson Veterinary, Greeley, CO), and ophthalmic ointment (Major Pharmaceuticals, Indianapolis, IN) was applied to each eye. The right hindlimb was shaved and cleaned with betadine/ethanol scrubs. Once clean, a 1 cm incision was made laterally over the right upper hindlimb, and blunt dissection was carried down to expose the femur and strip the muscle in the diaphyseal region. Next, the knee was flexed, and a sterile Dremel rotary cutting tool (DREMEL, Racine, WI) was used to cut the femoral diaphysis. A 25-gauge needle was used to split the patellar tendon and the needle was then manually advanced between the femoral condyles into the femoral intramedullary canal and through the greater trochanter. To stabilize the femur, the needle was bent onto itself and was pulled in an anterograde direction tautly against the greater trochanter. Type I collagen membranes (RCM6 Resorbable Collagen Membrane, ACE, Brockton, MA) were cut into 2 cm x 1 cm strips, treated with either saline (control) or seeded with neonatal BMECs (1x10^6^ cells/membrane) from neonatal mice, and placed around the femoral diaphysis. The membrane was fixed into place with a 3-0 polyglycolic acid suture (J215H, Ethicon, Somerville, NJ). Muscle tissue and skin were then closed with 6-0 polyglycolic acid suture and standard 7 mm wound clips (RF7CS, Braintree Scientific, Braintree, MA), respectively. X-ray images were used to confirm alignment of the pin at the time of surgery and then were taken every week for 4 weeks to monitor bone healing. Mice were monitored twice daily for 5 days following the surgery. During this time, as previously detailed (30), cages were placed on water-heated pads and were also provided 0.5mL of saline (31). Mice were euthanized 4 weeks post-surgery and femurs were harvested for micro-computed tomography (µCT) and histological analysis.

### Isolation of endothelial cells from lungs and bone marrow

All tissue isolation was performed in a sterile environment. LECs and BMECs were isolated from neonatal mice (2-6 days of age) as described previously (4, 32, 33).

For neonatal LEC, lungs were finely minced into small pieces and then digested with 225 U/ml collagenase type 2 solution (Worthington Biochemical Corporation, Lakewood, NJ) for 1 hour at 37°C and 5% CO_2_. Simultaneously, Dynabeads™ Biotin Binder (Thermo Fisher Scientific, Waltham, MA) were conjugated with biotin rat anti-mouse CD31 antibody (BD Pharmingen™, San Jose, CA). The beads were washed three times with 0.1% BSA/PBS, mixed with 10 µg of CD31 antibody, and incubated while shaking at room temperature for 1 hour. The minced tissue was filtered through a 70 µM mesh strainer and the cell suspension was incubated with the CD31-conjugated Dynabeads for 1 hour at 4°C. Later, CD31+ cells were separated by magnetic field using a Dynamag 2 (Thermo Fisher Scientific, Waltham, MA). Isolated cells were plated in collagen I coated 6-well plates (Corning^®^, Corning, NY) at a density of 3 x 10^5^ cells/mL in Endothelial Cell Growth Medium 2 (PromoCell, Heidelberg, Germany) supplemented with Growth Medium 2 SupplementMix (PromoCell, Heidelberg, Germany) and Penicillin-Streptomycin-Glutamine (Thermo Fisher Scientific, Waltham, MA). The media was changed every other day and the cells were subcultured once 70% confluence was reached, around 5 days after seeding.

For neonatal BMECs, femurs and tibias were harvested, stripped of soft tissue, and cut into small pieces, which were then placed in a crucible with 5mL of alpha-MEM media. The bone marrow was displaced from the femur fragments by loosely grinding the bone with a pestle. Cells displaced in the media were transferred to a 50 mL conical tube fitted with a 70 µm mesh filter. The cell solution was then centrifuged, and the remaining pellet was seeded in a 6-well plate (Thermo Fisher Scientific, Waltham, MA). The cells were cultured in Complete Endothelial Cell Growth Media (ScienCell, Carlsbad, CA) supplemented with 10% fetal bovine serum (FBS), Endothelial Cell Growth Supplement, and 1% Penicillin/Streptomycin (ScienCell, Carlsbad, CA). All BMECs were subcultured for use in experiments.

### Proliferation assay

Cells were seeded into two 96-well plates at 5x10^3^ cells/well and cultured for up to 2 days. Cells were fixed with 5% neutral buffered formalin (NBF) at room temperature for 20 minutes on either day 1 or day 2 and then stained with 0.05% crystal violet for 30 minutes. Later, cells were washed with water and left to dry at room temperature. Once completely dry, images were taken with EVOS^®^ FL Cell Imaging System and cell numbers were counted using ImageJ.1.52a software.

### Tube formation assay

Tube formation was assessed by Matrigel tube formation assay as previously described (32, 34). Matrigel basement membrane matrix (Corning^®^, Corning, NY) was polymerized in 96-well plates (50μl/well) at 37°C and 5% CO_2_ for 45 minutes. Neonatal LECs and BMECs were plated on the polymerized basement membrane matrix at a density of 10,000 cells/well, suspended in their specific growth medium. Cells were incubated at 37°C and 5% CO_2,_ and images were taken after 4 hours and 6 hours for lung and bone marrow cells, respectively. The parameters analyzed included the number of complete tubes and total tube length, measured manually by three independent double-blinded readers using the Simple Neurite Tracer plugin within the ImageJ.1.52b Fiji software.

### Wound migration assay

Sheet migration is a characteristic of ECs and occurs in damaged tissues (35). Neonatal BMECs and LECs were seeded in an Imagelock 96-well plate (Essen BioScience, Ann Arbor, MI) at a density of 8 x 10^4^ cells/well and grown for 24 hours until 100% confluence was reached. A wound was created in the middle of each well using an IncuCyte^®^ WoundMaker (Essen BioScience, Ann Arbor, MI). Images were taken at time 0, and consecutively every 2 hours for 48 hours at 10X magnification using the IncuCyte ZOOM^®^ Live-Cell Analysis System (Essen BioScience). Images were analyzed using the IncuCyte™ Scratch Wound Cell Migration Software (Essen BioScience). The parameters for migration assessed were relative wound density (%), wound confluence (%), and wound width (µm).

### Gene expression

Total RNA was isolated from cultured cells with TRIzol (Invitrogen Life Technologies, Carlsbad, CA). cDNA was prepared using the SuperScript^®^ III First-Strand Synthesis System for RT-PCR (Invitrogen Life Technologies, Carlsbad, CA). Quantitative PCR was performed using FastStart Universal SYBR Green Master (ROX) (Roche Diagnostics GmbH, Mannheim, Germany) as previously described (33). The following genes were analyzed: CD31 Antigen (CD31), Vascular Endothelial Growth Factor (VEGF), Fms Related Tyrosine Kinase 1 (Flk-1), Fms Related Receptor Tyrosine Kinase 1 (Flt-1), Angiopoietin 1 (ANGPT1), and Angiopoietin 2 (ANGPT2). 18S rRNA (18S) served as the internal control. The sequences of all the primers are included in **Table 1**. Relative gene expression was calculated using the 2^-ΔΔCT^ method.

**Table 1:** Quantitative PCR primers sequences.

**Genes**	**Orientation**	**Sequence (5′-3′)**
CD31	Forward	ACGCTGGTGCTCTATGCAAG
	Reverse	TCAGTTGCTGCCCATTCATCA
VEGF	Forward	GAGGTCAAGGCTTTTGAA
	Reverse	CTGTCCTGGTATTGAGGG
FLK-1	Forward	AGTTGGCAACGCAGGAAAAC
	Reverse	GGGATTGACTTTGCCCCAGT
FLT-1	Forward	CCACCTCTCTATCCGCTGG
	Reverse	ACCAATGTGCTAACCGTCTTATT
ANGPT1	Forward	CACATAGGGTGCAGCAACCA
	Reverse	CGTCGTGTTCTGGAAGAATGA
ANGPT2	Forward	CCTCGACTACGACGACTCAGT
	Reverse	TCTGCACCACATTCTGTTGGA
18S	Forward	CGCCGC-TAGAGGTGAAATTC
	Reverse	CGAACCTCCGACTTTCGTTCT

### Tissue processing and histology

Femurs were excised and fixed overnight in 10% neutral buffered formalin (NBF, Thermo Fisher Scientific, Waltham, MA), transferred to 70% EtOH, and stored at 4°C. Once µCT scans were completed (see below), femurs were placed in Immunocal Decalcifier (Satlab, Mckinney, TX) for demineralization for 24-48 hours at 4°C. Next, the bones were dehydrated through a series of concentrations of ethanol, cleared in xylene, infiltrated with paraffin, and finally, embedded in fresh paraffin. Five-micrometer-thick longitudinal sections were cut using a rotary microtome. Two sections per specimen were dewaxed and stained with Alcian Blue and Picrosirius Red to better visualize the fracture site. Histomorphometric analysis was performed using the Osteomeasure high-resolution digital video system software (Osteometrics Inc., Decatur, GA, USA). Callus area, bone area, cartilage area, and fibrous area were measured, and the percentage of bone, cartilage, and fibrous area was calculated.

### Micro-computed tomography imaging

Femurs were imaged using a desktop SkyScan 1172 µCT imaging system (SkyScan, Kontich, Germany, 60 kV, 5.9 µm voxel). The fractured bones were scanned (60kV, 6 µm resolution, 4000 pixels). The images were reconstructed using NRecon v.1.7.3. (Dyanmic Range for reconstruction was set for 0-0.1). Reconstructed images were used to build three-dimensional images using Bruker CT-Analyzer (v.1.15 CTAn). For facture callus analysis, the ROI was centered at the fracture site and extended toward the callus end in each direction. Reported variables include percent bone volume (BV/TV), trabecular number (Tb.N), trabecular thickness (Tb.Th), and trabecular separation (Tb.Sp),

### Statistical analysis

All data are reported as mean ± standard deviation of the mean. Statistical analyses were performed using GraphPad Prism version 8.0.0 for Windows (GraphPad Software, San Diego, CA). Unpaired two-tailed t-tests assuming unequal variances were performed to compare neonatal LECs to BMECs in tube formation path length and number, as well as gene expression for all genes. Paired two-tailed t-tests with assumed unequal variances were completed to compare all three wound migration parameters. Two-way ANOVA was done to compare neonatal LEC and BMEC timepoints and the groups’ proliferative capacity. Histological data were analyzed by using a non-parametric test. Significance was determined as p<0.05.

## Results

### Proliferation of neonatal lung and bone marrow endothelial cells

**Figure 1:**
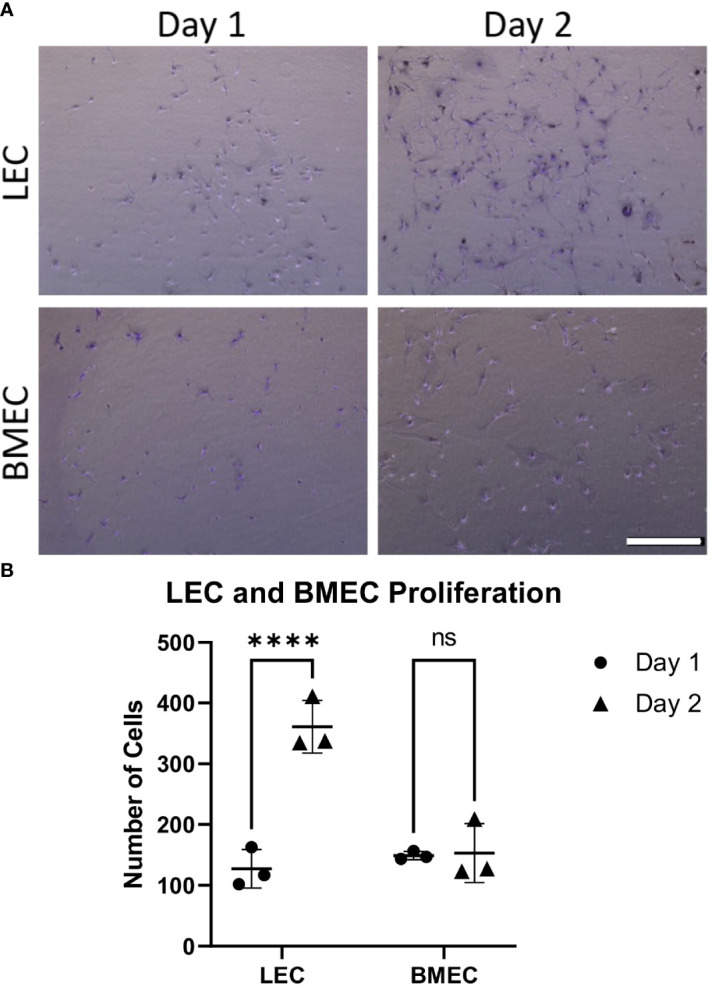
Neonatal BMEC and LEC proliferation assays. Neonatal BMEC and LEC cultures were stained with crystal violet and counted using ImageJ. **(A)** Crystal violet-stained representative images for Day-1 and -2 for both neonatal LEC and BMEC proliferation assays. **(B)** Cell counts of Day-1 and -2 for both LEC and BMEC populations show differences between Day 1 and Day 2. **** p ≤ 0.0001; (n = 5 biological replicates in triplicate/group); ns, not significant. Scale bar = 500µm.

Cell proliferation was examined by comparing the counts between two time points, one day after seeding and two days after seeding. While neonatal LECs showed a significant increase in cell number over two days, neonatal BMECs did not show a significant difference between day-1 and day-2. This suggests that neonatal BMECs have a lower proliferative ability within the timeframe used when compared to neonatal LECs (**Figure 1**).

### Tube formation analysis of neonatal lung and bone marrow endothelial cells

**Figure 2:**
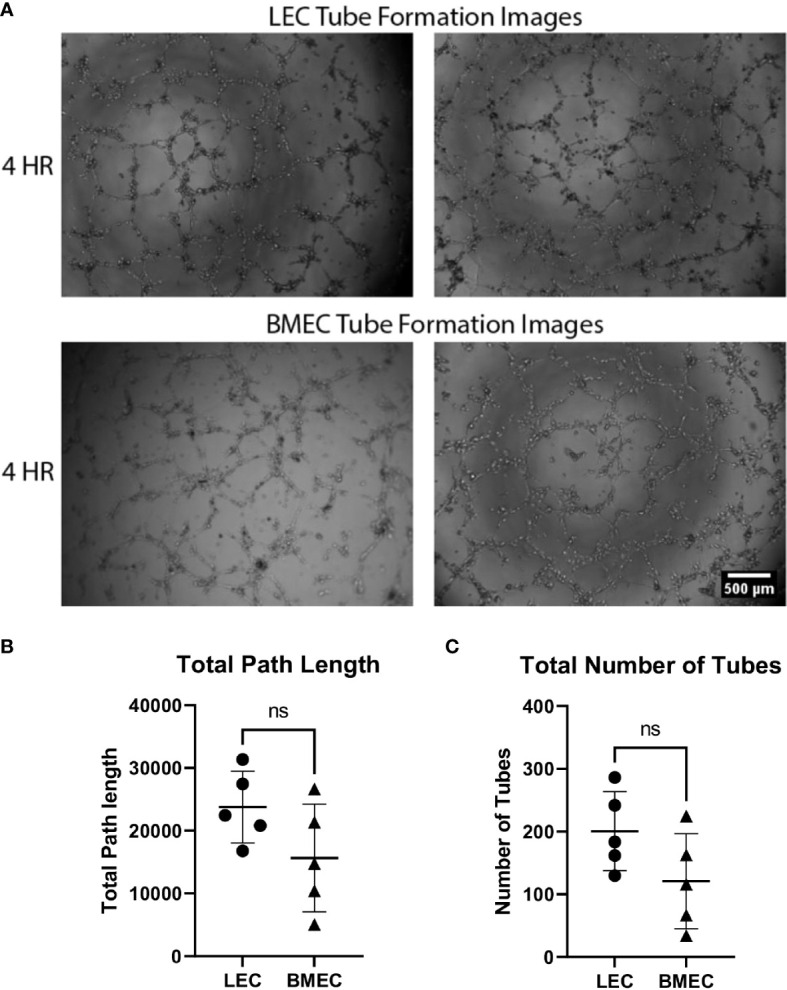
Neonatal BMEC and LEC tube formation on Matrigel. Neonatal LECs and BMECs were seeded on Matrigel basement membrane at a 1x10^4^cell density/100uL of respective media. These images were captured at four hours of incubation. Images were analyzed using ImageJ to trace tube networks, ultimately determining the number of tubes and the lengths of individual tubes. **(A)** Two images for both neonatal LEC and BMEC tube formation assays captured at 4 hours. **(B)** Total path lengths of neonatal LECs and BMECs. **(C)** Total number of tubes for neonatal LECs and BMECs. Data are expressed as mean ± SD (n = 5 replicates in triplicate/group). ns, not significant.

The angiogenic potential of both neonatal LECs and neonatal BMECs was evaluated by the formation of tubes. Cells began forming tube networks at around 2 hours post-seeding and were imaged at 4-, 6-, and 8-hours post-seeding. It was observed that tubes peaked around 4 hours and gradually broke down thereafter. There were no significant differences observed between the two cell groups for either the number or the length of the tubes, suggesting that both LECs and BMECs have similar vessel forming abilities (**Figure 2**).

### Migration of neonatal lung and bone marrow endothelial cells

**Figure 3:**
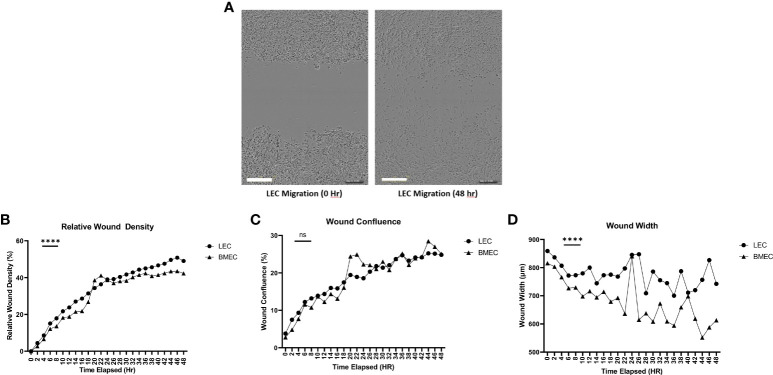
Neonatal BMEC and LEC wound migration *via* scratch assay. Neonatal BMECs and LECs were seeded in 96 well plates at 1x10^5^ cells/well. A scratch was made across the plate **(A)**. Images were obtained every 2 hours for 48 hours. **(B)** Relative Wound Density, **(C)** Wound Confluence, and **(D)** Wound Width were analyzed. ***P ≤ 0.001 between neonatal LEC and BMEC; (n = 5; LEC/n = 3; BMEC), Scale Bar = 300µm. ns: Not significant, P > 0.05

Wound migration was determined using three parameters. Relative wound density compares the concentration of cells inside the wound to the concentration of cells outside the wound area. This parameter is used as a self-normalizing tool to observe changes in the cell density outside the wound created by proliferation and/or pharmacological effects (36). Neonatal LECs had a significantly higher relative wound density (p<0.0001) than neonatal BMECs. The second parameter examined was the wound confluence, which is the cell density within the wound area. There was no significant difference in wound confluence between neonatal LEC and neonatal BMEC. Finally, wound width measures the distance between the wound edges as it closes. Neonatal BMECs showed a significantly better migratory ability (p<0.0001) than neonatal LECs. These data suggest that both neonatal LECs and neonatal BMECs showed an increase in migratory activity in different ways (**Figure 3**).

### Angiogenic gene expression

**Figure 4:**
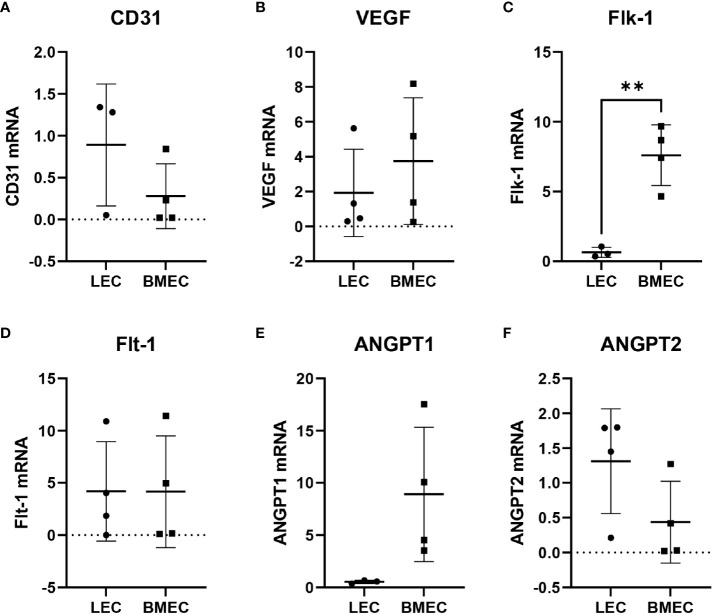
Relative gene expression of **(A)** CD31, **(B)** VEGF, **(C)** Flk-1, **(D)** Flt-1, **(E)** ANGPT1, and **(F)** ANGPT2. RNA was isolated from neonatal LECs and neonatal BMECs using the TRIzol method, and then cDNA was prepared using SuperScript^®^ III First-Strand Synthesis System for RT-PCR. Finally, quantitative PCR was carried out. Ct values were determined for all the genes including 18S as the internal control. The 2^-ΔΔC^_T_ method was used to calculate relative gene expression in fold change. **P ≤ 0.01 (n = 3 replicates in triplicate/group).

Since neonatal ECs were isolated from two different tissues using different methods, six different angiogenic-related genes were examined to determine whether differences in their gene expression exist. The only significant differences observed were with Flk-1. Neonatal BMECs had significantly higher (p=0.0066) mRNA levels of Flk-1 in comparison to neonatal LECs. The other five genes, CD31, VEGF, Flt-1, ANGPT1, and ANGPT2, did not exhibit significant differences in their expression between the two cell groups, suggesting a mostly similar gene expression profile between neonatal LECs and neonatal BMECs (**Figure 4**).

### Femur fracture study

**Figure 5:**
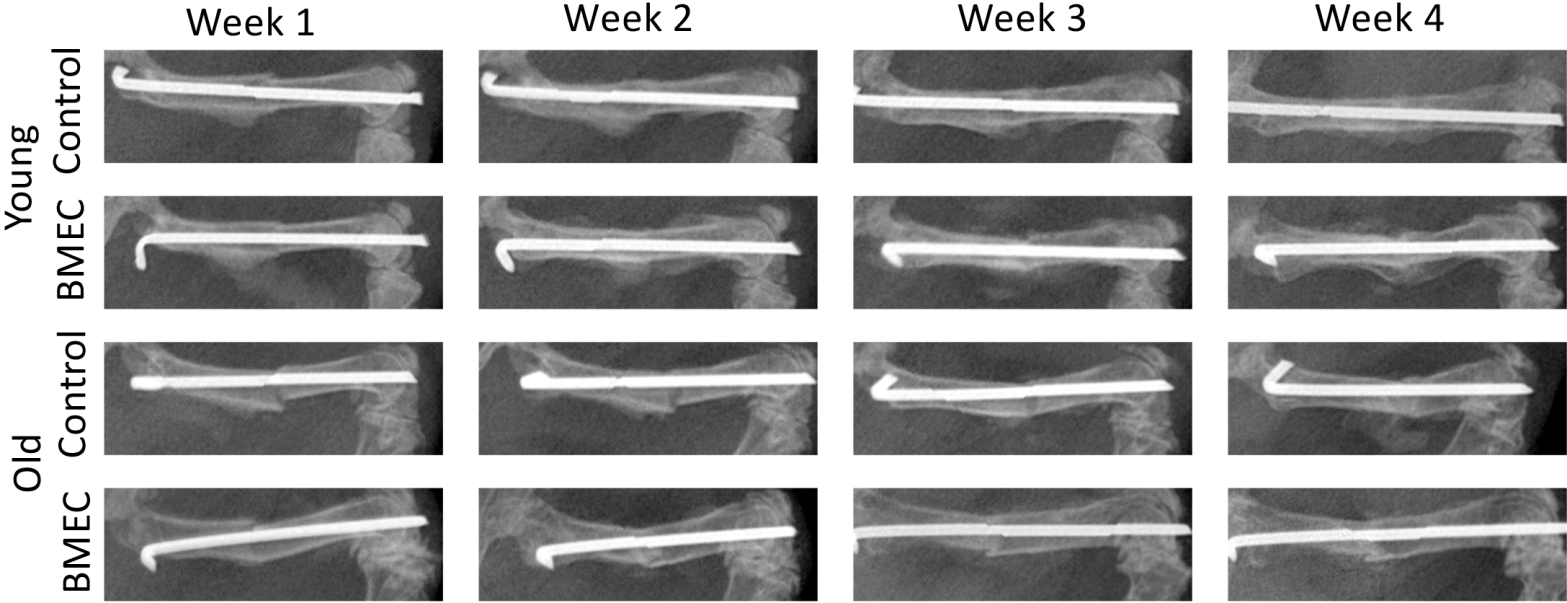
X-ray images of fracture in both young and old mice treated with either saline or neonatal BMECs weekly after fracture surgery. Callus formation was seen earlier in young mice compared to old mice. Compared to saline, treatment with neonatal BMECs seemed to induce larger callus.

X-rays were taken at baseline and once a week for four weeks post-surgery. **Figure 5** shows the representative X-rays and changes in callus formation over the four-week period. Callus formation was seen earlier in young mice compared to aged mice. Compared to saline, treatment with neonatal BMECs seemed to induce a slightly larger callus.

**Figure 6:**
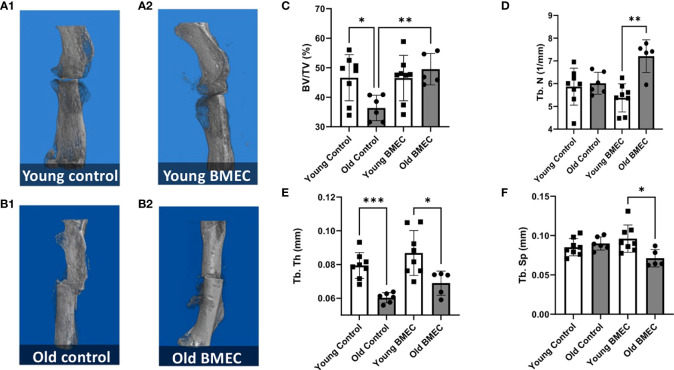
Femurs were harvested four weeks after the fracture surgery. µCT imaging was performed. The representative 3D model images of fractured femurs are shown in **(A.1)** young mice treated with saline, **(A.2)** young mice treated with neonatal BMECs, **(B.1)** old mice treated with saline, and **(B.2)** old mice treated with neonatal BMECs. Several callus parameters were analyzed within the fracture region of the reconstructed μCT images, including: **(C)** mineralized callus volume (BV/TV), **(D)** trabecular number (Tb.N), **(E)** trabecular thickness (Tb.Th), and **(F)** trabecular separation (Tb.Sp). Data points represent individual mice. * P ≤ 0.05, **P ≤ 0.01, ***P ≤ 0.001.

**Figure 6** shows the µCT images of young (A) and aged (B) mice treated with neonatal BMECs and saline controls, respectively and quantitated µCT data. The µCT data analysis shows that implantation of neonatal BMECs to the fracture site significantly increased callus BV/TV and Tb.N in aged mice when compared to aged mice implanted with only collagen sponge. However, BMECs did not improve callus BV/TV in young mice. These data suggest a positive effect of neonatal BMECs on fracture healing in aged mice.

**Figure 7:**
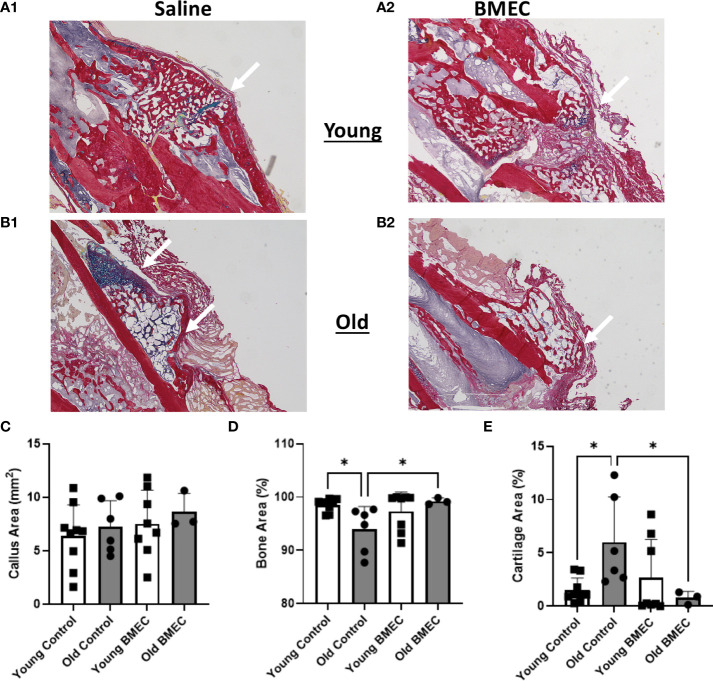
Histomorphometry at four weeks after fracture. Histological sections stained with picrosirius red (bone) and alcian blue (cartilage, arrows) are shown in **(A.1)** young mice treated with saline, **(A.2)** young mice treated with BMECs, **(B.1)** old mice treated with saline and **(B.2)** old mice treated with BMECs. Quantitative analysis describes **(C)** callus area, **(D)** percentage of bone area and **(E)** percentage of cartilage area. Data points represent individual mice. *P ≤ 0.05.

Histological analysis of the fractured femurs showed no significant difference in the size of the callus between BMEC treated groups when compared to their controls (**Figure 7**). Further analysis of callus composition indicates percentage of bone area in the aged control group is significantly less than the young control group, whereas the percent cartilage area in the aged control group is significantly greater than the young control group. These data suggest the endochondral ossification is significantly delayed in aged mice. Further, the aged mice treated with neonatal BMECs showed a significant improvement in the healing process when compared to the aged controls. Aged mice treated with BMECs showed a significantly higher amount of bone (99.28%) than the saline-treated control (93.99%) and a significantly less amount of cartilage within the callus. These data suggest that neonatal BMECs have positive effects on bone healing, especially neonatal BMECs significantly enhanced fracture repair in aged mice.

## Discussion

The rate of bone loss accelerates with aging, often resulting in osteoporosis or osteopenia, making the elderly at a higher risk for fractures (37). There are currently many viable drug options used for treatment against osteoporosis, however, many of these drugs, including the most commonly used class of drugs, bisphosphonates, act by inhibiting resorption, which disrupts bone remodeling and suppresses *de novo* bone formation, interfering with fracture healing (38). Bisphosphonates, however, can have negative effects on the vasculature and other organs (39). FDA approved bone healing agent, bone morphogenetic protein 2 (BMP-2), stimulates bone healing. Unfortunately, BMP-2 is associated with some side effects, including heterotopic bone formation and an increased cancer risk (40–43). Therefore, there is still a need to identify new treatments to improve fracture healing but reduce side effects. Therapies that target angiogenesis/vascular invasion, including cell therapies examined here, may prove promising.

Indeed, vascular invasion at the fracture site is considered an essential step in the healing process (24, 39, 44), and EPCs have been shown to directly assist in that through enhancing angiogenesis and vasculogenesis (11, 14, 21, 24, 45). Since the initial discovery of EPCs, their potential use for regenerative medicine has laid the foundation for new research topics, leading to some ambiguity surrounding EPCs with researchers defining multiple subtypes and tissue origins while using varying methods of isolation. Some of the major origin sites for EPCs are peripheral blood, umbilical cord blood, bone marrow, and tissue-resident cells in vasculature including within lung tissue (11, 15–17). Heterogeneity in the vasculogenic potential of EPCs has also been observed and thus leads to a widescale of variance in engraftment potential of EPCs to host vasculature (16); for this reason, we isolated both LECs and BMECs from neonatal tissues, investigated and compared the traits of these cells *in vitro*.

The choice of using progenitor cells instead of stem cells was made due to the fact that progenitor cells are already fated to differentiate down a certain lineage, whereas stem cells could differentiate away from the desired cell line (46). Using neonatal cells was another important detail, since neonatal mesenchymal stem cells (MSCs) have displayed superior proliferative ability, less susceptibility to mutation, and lower immunogenicity, compared to adult derived MSCs (46–48). Another significant advantage to neonatal EPCs is that they are derived from non-invasively obtained tissues such as cord blood or the placenta (48).

Neonatal BMECs were isolated by crushing bone marrow out of femurs and then seeding those cells in an EPC-specific media, while neonatal LECs were isolated using CD31 biotin magnetic beads to separate them into a homogenous cell culture. We performed tube formation analysis, proliferation, and wound migration experiments on the cells. In culture, neonatal LECs are highly proliferative, contain specific markers, specifically CD31 for our experiment, and develop vasculature-like networks. ECs derived from young mice typically take around two weeks to appear in culture (19, 49), but the neonatal LECs isolated in this study appeared a day or two after isolation. Moreover, they expanded very rapidly, and both neonatal LECs and neonatal BMECs were ready for subculture 5 days post-isolation.

Neonatal LECs and neonatal BMECs displayed similar trends in all categories analyzed, except for their ability to proliferate. Over two days, neonatal LECs were significantly more robust proliferators than neonatal BMECs. Although our experiment was not technically a proliferation assay, the cells were seeded at low densities and then given two days to grow, and comparative counts were made of cells between day-1 and -2, serving as a reliable proxy for proliferation. While neonatal LEC expansion involved the forming of colonies that then expanded until the plate was confluent, neonatal BMECs covered the plate randomly and proliferated thereafter. Tube formation analysis, which displays the functional potential of ECs, showed comparable results between neonatal LECs and neonatal BMECs for both the number of vessel-like tubes formed and for the total length of each path. These data suggest that both cell types have a similar angiogenic potential. Additionally, the migration potential of these ECs was tested. This was important in showing that the cells could migrate into damaged tissue, as would be needed if the cells were added to a tissue scaffold. Our findings showed that while neonatal LECs migrated to the inside of the wound at a higher density than neonatal BMECs, neonatal BMECs were significantly better at migrating than neonatal LECs, lending further support to BMEC use in bone regeneration.

Our data showed that neonatal BMECs had significantly higher Flk-1 expression than neonatal LECs. Flk-1, also known as VEGF receptor 2 (VEGFR2), is a tyrosine kinase receptor that binds VEGFs. Flk-1 activation influences angiogenesis and vasculogenesis through EC proliferation, migration, promoted survival, and differentiation (50). Flk-1 has a lower affinity for binding VEGF-A, the major pro-angiogenic VEGF, but much higher signaling activity than VEGF receptor 1 (VEGFR1) once bound (50, 51). Despite having significantly higher Flk-1, there was no significant difference in Flt-1 between the cell types. Neonatal LECs are still reactive to VEGF signaling; however, neonatal BMECs higher expression of Flk-1 suggests they more actively influence blood vessel development.

Neonatal LECs are isolated specifically with CD31, a requisite EC marker, making their population homogenous, while neonatal BMECs are comprised of a heterogeneous population that may be composed of stalk and tip cells, ECs, parenchymal cells, and endothelial cell-like cells (33). These differences may influence the cells performance in the assays tested. Pericytes, important for blood vessel formation in the central nervous system for example, have been shown to act as vasculature progenitor cells, promote new vessel formation, and recruit other pro-angiogenic cells in models of myocardial ischemia (46). Historically, most EPCs have been isolated from bone marrow and blood. More recently, vasculature and tissue resident EPCs have come into the picture. Better understanding the stem cell niche for EPCs and MSCs within vasculature and tissue could be very important to providing information on ischemic tissue repair and a way to collect the most potent EPCs for use in tissue regeneration.

Our characterization tests showed both neonatal BMECs and neonatal LECs propensity to form vessel-like structures, to proliferate *in vitro*, and to migrate efficiently. Along with that, cells can be harvested quickly, and the isolation methods yield strong populations of cells that expand expediently, all suggesting that both cell populations remain a strong choice for tissue regeneration. However, use of BMECs is more feasible for clinical applications than LECs. In addition, one previous study has shown that BMECs derived from femurs and tibias have been used to improve fracture healing and callus formation of critical size defects in adult rats (24), while human BMECs were shown to induce vasculogenesis in myocardial infarct scars, reducing scar tissue, and keeping myocardial tissue functional, improving ventricular function in a rodent myocardial ischemic model (52). Here, we tested neonatal BMECs ability to improve bone healing in a femur fracture in aged mice.

In this study, the µCT imaging analysis shows the percent of mineralized bone tissue (BV/TV) in aged mice implanted with BMECs in collagen sponge is significantly higher than the aged mice implanted with only collagen sponge. Consistent with the µCT data, the histological analysis of callus composition demonstrated that aging slowed down the process of endochondral ossification as evidenced by the significantly greater percentage of cartilage area in the callus in aged control mice in comparison with the young controls. While neonatal BMECs did not improve endochondral ossification in young mice, the cells significantly reduced the percentage of cartilage area in callus in aged mice. These data suggest that treatment with neonatal BMECs was able to rescue slow progress of bone healing in aged mice.

BMECs can act directly upon the vasculature by differentiating and incorporating into the tissue to grow the vasculature but appear to operate more in a paracrine role (19), meaning they release growth factors, such as VEGF, that signal the residential endothelium to form new vessels. In this study, it is possible that the neonatal BMECs were indeed successful at engrafting to the injured region, but the residential ECs were unable to properly respond to the paracrine signaling. This may be even more prevalent in the aged mice, where the cells would have senescence-derived inefficiencies. Mutations build up steadily in adult stem cells over time, which could potentially lead to phenotypic changes that disrupt regenerative abilities (53). To formally test this idea, implantation of GFP labeled BMECs would allow for *in vivo* stem cell tracking of BMEC engraftment at the fracture site. In addition, recent reports have suggested that extracellular vesicles, such as exosomes, secreted by EPCs enhance bone healing potentially by participating in cell-to-cell communication and stimulating angiogenesis (39, 45, 54–56). Future studies are required to better characterize exosomes secreted from EPCs as well as examine the role of EPCs derived exosomes in bone healing in aging conditions.

In conclusion, the *in vitro* characterization of neonatal LECs and neonatal BMECs showed similar propensity for tube formation, proliferation, and migration, all needed for proper vessel development to affect tissue regeneration. This study also showed that while aging delayed endochondral bone formation during bone healing, treatment with neonatal BMECs improved endochondral bone healing in aged mice such that it was indistinguishable from that observed in young control healing. Altogether, our data supports the notion that BMECs could serve as a potential treatment to improve angiogenesis and tissue regeneration in aged fracture healing.

## Data availability statement

The original contributions presented in the study are included in the article/supplementary material. Further inquiries can be directed to the corresponding author.

## Author contributions

MM analyzed data, performed histology and wrote the paper. CV and JL performed cell culture and animal studies. MK, JL, UD, and CV designed research, analyzed, and interpreted data. UD, OA, SM, and RB assisted with tissue collection and animal experiments. AK, AM, and HB measured tube assay date. MN measured proliferation data. All the authors approve of the final version of the paper and take responsibility for the contents of the paper. JL and MK take responsibility for the integrity of the data analysis.

## Funding

This project was supported by NIH R01 AG060621 (MK and JL) and by the Cooperative Center of Excellence in Hematology (CCEH) Award funded by NIH U54 DK106846 (MK). This work was also supported by an Indiana University Collaborative Research Grant (MK, JL). This material is also the result of work supported with resources and the use of facilities at the Richard L. Roudebush VA Medical Center, Indianapolis, IN: VA Merit #BX003751(MK). Disclaimer: The views expressed in this article are solely those of the authors and do not necessarily represent the official position or policy of any of the aforementioned agencies.

## Acknowledgments

We would like to thank the Indiana Center for Musculoskeletal Health Histology Core for assisting with histological preparation and the Indiana University Simon Cancer Center Angiogenesis Core for assisting with the wound migration assays.

## Conflict of interest

The authors declare that the research was conducted in the absence of any commercial or financial relationships that could be construed as a potential conflict of interest.

## Publisher’s note

All claims expressed in this article are solely those of the authors and do not necessarily represent those of their affiliated organizations, or those of the publisher, the editors and the reviewers. Any product that may be evaluated in this article, or claim that may be made by its manufacturer, is not guaranteed or endorsed by the publisher.
